# Weighing the unknowns: Value of Information for biological and operational uncertainty in invasion management

**DOI:** 10.1111/1365-2664.13904

**Published:** 2021-06-22

**Authors:** Shou‐Li Li, Joseph Keller, Michael C. Runge, Katriona Shea

**Affiliations:** ^1^ Department of Biology The Pennsylvania State University University Park PA USA; ^2^ State Key Laboratory of Grassland Agro‐Ecosystems Center for Grassland Microbiome, and College of Pastoral, Agriculture Science and Technology Lanzhou University Lanzhou People’s Republic of China; ^3^ US Geological Survey Eastern Ecological Science Center at the Patuxent Research Refuge Laurel MD USA

**Keywords:** biocontrol, biological uncertainty, *Carduus nutans*, decision‐making, invasion management, matrix model, operational uncertainty, Value of Information

## Abstract

1. The management of biological invasions is a worldwide conservation priority. Unfortunately, decision‐making on optimal invasion management can be impeded by lack of information about the biological processes that determine invader success (i.e. biological uncertainty) or by uncertainty about the effectiveness of candidate interventions (i.e. operational uncertainty). Concurrent assessment of both sources of uncertainty within the same framework can help to optimize control decisions.
2. Here, we present a Value of Information (VoI) framework to simultaneously analyse the effects of biological and operational uncertainties on management outcomes. We demonstrate this approach with a case study: minimizing the long‐term population growth of musk thistle *Carduus nutans*, a widespread invasive plant, using several insects as biological control agents, including *Trichosirocalus horridus*, *Rhinocyllus conicus* and *Urophora solstitialis*.
3. The ranking of biocontrol agents was sensitive to differences in the target weed's demography and also to differences in the effectiveness of the different biocontrol agents. This finding suggests that accounting for both biological and operational uncertainties is valuable when making management recommendations for invasion control. Furthermore, our VoI analyses show that reduction of all uncertainties across all combinations of demographic model and biocontrol effectiveness explored in the current study would lead, on average, to a 15.6% reduction in musk thistle population growth rate. The specific growth reduction that would be observed in any instance would depend on how the uncertainties actually resolve. Resolving biological uncertainty (across demographic model combinations) or operational uncertainty (across biocontrol effectiveness combinations) alone would reduce expected population growth rate by 8.5% and 10.5% respectively.
4. *Synthesis and applications*. Our study demonstrates that intervention rank is determined both by biological processes in the targeted invasive populations and by intervention effectiveness. Ignoring either biological uncertainty or operational uncertainty may result in a suboptimal recommendation. Therefore, it is important to simultaneously acknowledge both sources of uncertainty during the decision‐making process in invasion management. The framework presented here can accommodate diverse data sources and modelling approaches, and has wide applicability to guide invasive species management and conservation efforts.

## Graphical abstract

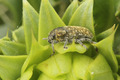

## 1 INTRODUCTION

Biological invasions cause serious harm around the globe. They reduce native biodiversity, alter ecosystem structure and functioning and cause enormous economic loss (Novak, 2007; Buckley & Han, 2014; Buckley & Csergo, 2017). Unfortunately, uncertainty about the key processes that drive invader success hampers decision‐making about effective interventions. Identifying which uncertainties most strongly influence management outcomes can facilitate the decision‐making process by allowing researchers to prioritize research to resolve such key uncertainties (Runge, Converse, & Lyons, 2011).

Invasion ecologists have frequently evaluated the efficacy of management actions based on perturbation analyses (Jongejans et al., 2008; Caswell & Sánchez Gassen, 2015). Perturbation analyses quantify the potential effect of a small change in a life‐history component (e.g. survival, growth or fecundity) on the overall population performance (e.g. long‐term population growth; Ramula, Knight, Burns, & Buckley, 2008; Caswell & Sánchez Gassen, 2015). Highly sensitive life‐history transitions are then recommended as targets for management interventions (Kroon, van Groenendael, & Ehrlén, 2000; Ramula et al., 2008). However, highly sensitive transitions may also be the ones with the smallest range of variation achievable by management (Baxter, McCarthy, Possingham, Menkhorst, & McLean, 2006). For example, reduction in adult survival might contribute most to reduced population growth of woody invaders (Koop & Horvitz, 2005; Li & Ramula, 2015), yet reducing the survival rate of adult woody plants may be least feasible in practice (Baxter et al., 2006).

Uncertainties about the biology of invaders and about the effectiveness of interventions are rife, especially for novel invaders and situations where data are limited. Biological uncertainties arise when invasive organisms' demographic processes such as survival, growth and fecundity are poorly understood. These uncertainties are hereafter referred to as biological uncertainty, but are also known as model, parametric or structural uncertainty in ecological settings (Nichols, Johnson, & Williams, 1995; Williams & Brown, 2016; Milner‐Gulland & Shea, 2017). Operational uncertainties arise due to limited information on intervention effectiveness, logistical constraints, feasibility or cost (Clewley, Eschen, Shaw, & Wright, 2012). Operational uncertainty is also referred to as partial controllability in ecological decision theory (Williams & Johnson, 1995). Studies to examine the effectiveness of a management action in practice are often lacking. When effectiveness is evaluated, it is often provided as a single value obtained from one study (Shea, Kelly, Sheppard, & Woodburn, 2005; Shea, Sheppard, & Woodburn, 2006; Li et al., 2013). However, ignoring uncertainty in control effectiveness and applying the same effectiveness value to guide management in different invasion contexts can be problematic, as a control action effective in one situation may be less so in another (Shea et al., 2005).

Here, we demonstrate how these two sources of uncertainty, biological and operational, together influence management decisions. We use an illustrative case study focused on the selection of the most appropriate biological control agent(s) to control musk thistle *Carduus nutans* L., a widespread invasive plant species. In this study, biological uncertainty refers to uncertainty about *C. nutans*' demographic processes, as represented by demographic models, while operational uncertainty refers to uncertainty about the effectiveness of biocontrol agents to control the thistle, similar to the distinction drawn by Dodd, Ainsworth, Burgman, and McCarthy (2015). Both sources of uncertainty are epistemic (i.e. arising from a lack of knowledge about the state of a system that can be reduced through learning) as opposed to aleatory (i.e. arising from environmental variation and other uncontrollable stochastic events, which usually cannot be reduced through learning; Regan, Colyvan, & Burgman, 2002; Shea, Tildesley, Runge, Fonnesbeck, & Ferrari, 2014). Biocontrol has been widely applied to control this weed, and efforts have resulted in varied success (Shea et al., 2005). Multiple models for this species' demography in both native and invasive populations exist, and corresponding information describing effectiveness of several different biocontrol agents is available (McCarty & Lamp, 1982; Woodburn, 1997; Shea & Kelly, 1998). To assess biological and operational uncertainties within the same model structure, we use Value of Information (VoI) analysis. VoI analyses address how uncertainty affects decision‐making, conceptually similar to the way that elasticity analyses address how uncertainty affects ecological processes (Felli & Hazen, 1998). Like elasticity analysis, VoI analysis is a sensitivity analysis—it measures the effect of uncertainty; but unlike elasticity analysis, VoI analysis focuses on the relevance of uncertainty *to the decision maker*, rather than to the scientist. The Value of Information measures how much the outcome of the decision could be improved if uncertainty were resolved before committing to action (Runge et al., 2011). We first demonstrate VoI's use for addressing biological uncertainty alone, and then extend the approach to include both biological and operational uncertainties. We discuss the application of our framework to biocontrol of *C. nutans* in particular, to biocontrol programmes in general and to any decision‐making process in invasion management. In short, this approach can be applied in a wide range of environmental management programmes where both biological and operational uncertainty impedes the choice of management.

## 2 MATERIALS AND METHODS

### 2.1 Study system

Musk thistle (*Carduus nutans* L.: Asteraceae) is a short‐lived monocarpic herbaceous plant. Its seeds germinate in fall or spring, and individuals form rosettes and grow for one to several years before bolting and flowering. Musk thistle produces flower heads 1.5–4.5 cm in diameter, and flower heads can hold up to 1,500 achenes (Desrochers, Bain, & Warwick, 1988). Plants die after reproduction (Desrochers et al., 1988). This species originated in Eurasia, but has invaded many regions, including Argentina, Australia, New Zealand and North America, and it grows in pastures, croplands, roadsides and disturbed areas (Desrochers et al., 1988). Musk thistle's spiny leaves and stems prevent livestock from grazing, reducing pasture productivity (Desrochers et al., 1988). It is therefore commonly listed as a noxious weed in its invaded ranges. Three main biocontrol agents have been released for musk thistle management. Its rosettes are attacked by the root‐crown weevil *Trichosirocalus horridus* Panzer; developing flower heads are attacked by larvae of the receptacle weevil *Rhinocyllus conicus* Froel. and the receptacle gallfly *Urophora solstitialis* L. (McCarty & Lamp, 1982; Woodburn, 1997; Shea & Kelly, 1998).

### 2.2 Capturing biological and operational uncertainty

We present a decision context in which a biocontrol practitioner is deciding among a suite of interventions to manage *C. nutans*. We assume that the ***management objective*** is to minimize the long‐term population growth rate (*λ*) of *C. nutans*. We first calculated baseline *λ* under no action. Each of the three biocontrol agents can be released alone, or together with other agents in an Integrated Pest Management framework. Thus, there are seven ***alternative control actions*** or ***action combinations***: *T*. *horridus alone, R. conicus alone, U. solstitialis alone, T. horridus & R. conicus together, T. horridus & U. solstitialis together, R. conicus & U. solstitialis together and all three insects together*.

We used matrix projection models (Shea & Kelly, 1998; Jongejans et al., 2008) as our ***modelling framework*** to predict the effect of each of the seven alternative actions in achieving the management objective. Matrix projection models allow us to project the invasion trajectory (population growth rate *λ* > 1 means the population is invading), conduct elasticity analyses to determine which life cycle transitions may be vulnerable to management, and evaluate potential interventions by changing components (single or multiple elements or vital rates) of the matrix to examine the effects on *λ*. In our matrix models, individuals in a population are classified into four stages: seed bank (SB), small rosettes (S) with less than 20% chance of surviving and flowering in the next year, medium rosettes (M) with between 20% and 80% chance of surviving and flowering in the next year and large rosettes (L) with greater than 80% probability of surviving and flowering in the next year (Figure S1; Shea et al., 2005; Jongejans et al., 2008). Four population matrices were constructed using data from four populations: one in the native range (a field site at La Cavalerie, a town in the south of France, 44°00′S, 3°08′E) and three in invaded ranges (Kybeyan, a field site at Kybeyan near Canberra in southern New South Wales, Australia, 36°22′S, 149°24′E; Midland, a rural site on the North Island, New Zealand, 40°15′S, 175°43′; and Kansas, a site at the University of Kansas' John H. Nelson Environmental Study Area in Jefferson County, Kansas, USA, 39°02′S, 95°12′E). More details on vital rates, equations used to construct matrix transition elements and final matrices can be found in supplementary tables (Tables S2–S7) and Jongejans et al (2008). A biocontrol practitioner with an invasion in a novel setting may not know which of these different populations best represents how the plant will grow in the new setting; thus, ***biological uncertainty*** is represented by these matrix models, particularly by differences in the vital rates used for constructing transition elements in the matrices.

Uncertainty about the effectiveness of control actions is an important ***operational uncertainty***. We took two approaches to investigating the effect of operational uncertainty on management selection: one considering all possible levels of effectiveness, and one informed by published biocontrol agent impacts. We first explored the effects of operational uncertainty by simulating *λ* under a full range of effectiveness levels for each model. In the simulations, we reduced all survival, growth or fecundity rates in each matrix by 0%–100%, in intervals of 20%. We then examined operational uncertainty about the effectiveness of biocontrol agents achieved in practice by conducting a literature survey on the Institute for Scientific Information Web of Knowledge and through the Google search engine, using the Latin names of our candidate biocontrol agents as search terms. We identified a total of 64 relevant studies in 17 publications, which reported the effectiveness of our biocontrol agents or agent combinations (Table S1). We simulated the observed range of biocontrol effectiveness for each biocontrol agent or combination of agents for each model by modifying the corresponding underlying vital rates in the matrix transition elements. We then estimated *λ* for each model by calculating the dominant eigenvalue of each transition matrix.

### 2.3 Estimating and comparing the importance of biological and operational uncertainty with Value of Information (VoI)

We employed VoI analysis to examine how much the management outcome could be improved by resolving biological and operational uncertainty before committing to action. This information can guide information collection strategies. If resolving uncertainty would improve outcomes, gathering information may be worthwhile, but if resolving uncertainty would not improve outcomes, further learning is unnecessary. We first conducted an Expected Value of Perfect Information (EVPI; Howard, 1966) analysis, a common type of VoI analysis that quantifies how much the management outcome could be improved by perfect information resolving all sources of uncertainty. We initially demonstrate how EVPI can be easily conducted by only considering biological uncertainty, assuming the control effectiveness is known, that is, there is no operational uncertainty. We use the biocontrol effectiveness information from Shea et al (2006) as a case study for illustration. In Shea et al (2006), each candidate biocontrol has one fixed effectiveness value: *R*. *conicus* reduces seed production by 30%, *T. horridus* reduces plant growth by 87% and *U. solstitialis* reduces seed production by 70%. Considering only biological uncertainty, EVPI can be calculated as

(1) $$\text{EVPI}=\left[\sum_{i=1}^{q}p_{i}\min_{a}\lambda_{a,i}\right]‐\min_{a}\sum_{i=1}^{q}p_{i}\lambda_{a,i}.$$

Here, *q* is the total number of models, *λ_a,i_* represents population growth rate projected under action *a* = 1, 2, …, *A* *(A* = 4, including no management or release of one of three biocontrol agents), by model *i* = 1, 2, …, *q* (*q* = 3; we only considered the three models in the invaded ranges, excluding the model from the native range, France, because biocontrol agents already exist there and cannot be added). Parameter *p_i_* is the weight associated with model *i* (i.e. the prior belief that model *i* is the true model; subject to the constraint that the *p_i_* sum to 1), and *min_a_* indicates the lowest *λ* across management actions. Therefore, on the right side of the equation, the first term is the mean of the minimum *λ* value under each model (cell in row 6, column 7 of Table 1). This value describes outcomes when research is conducted to determine which model most accurately reflects the demography of the population targeted for management. With this information, managers would be able to select the optimal management action for the correct demographic model (column 6 in Table 1). Because we do not yet know which model would be selected, we average the outcomes that would result if managers made optimal selections based on each model. The second term on the right side of the equation is the minimum of the mean *λ* values across all models (cell in row 7, column 7 of Table 1). This value describes outcomes when research is not conducted and decision makers make a decision in the face of uncertainty, that is, without determining which demographic model best describes their target population. Without this information, managers calculate how each management option performs under each model, then choose the agent that does best, on average, across models, even if this agent is not optimal in some cases (row 5 in Table 1).

**TABLE 1:** The value of resolving uncertainty in the management of *Carduus nutans* with different biocontrol agents. Expected Value of Perfect Information (EVPI) represents the improvement in management outcome in terms of reduction in population growth rate (*λ*) by resolving uncertainty perfectly. The uncertainty is represented by three different countries in invasive range, Australia, New Zealand and USA. The three candidate biocontrol agents are *Rhinocyllus conicus*, *Trichosirocalus horridus* and *Urophora solstitialis*. The biocontrol effectiveness level is fixed for each biocontrol agent, and the effectiveness information is from Shea et al (2006). Cells filled in lighter grey indicate the lowest *λ* across biocontrol agents under each model, and the average of the lowest *λ* in each model. Cells in darker grey indicate the average of *λ* under each biocontrol agent across models, and the minimum of the average *λ* across models. The averages assign equal weight to each of the three models

Models	No management	*R. conicus*	*T. horridus*	*U. solstitialis*	Lowest *λ*	
Australia	1.20	0.93	0.50	0.59	0.50	
New Zealand	2.68	2.14	2.47	1.31	1.31	
USA	1.75	1.50	0.72	1.07	0.72	
Average of *λ* across models	1.88	1.52	1.23	0.99		
Average of the lowest *λ* in each model						0.84
Minimum of the average *λ* across models						0.99
EVPI						0.15
Improvement in management outcome						14.9%

We extend the EVPI to simultaneously assess both biological and operational uncertainty, with operational uncertainty represented by variation in biocontrol effectiveness levels. EVPI is then calculated as:

(2) $$\text{EVPI}=\left[\sum_{i=1}^{q}\sum_{j=1}^{r}p_{i}p_{j}\min_{a}\lambda_{a,i,j}\right]‐\min_{a}\sum_{i=1}^{q}\sum_{j=1}^{r}p_{i}p_{j}\lambda_{a,i,j},$$

where *q* is the number of models, *r* is the combination of effectiveness levels of candidate biocontrol agent or agent combination (hereafter referred to as effectiveness combination) and *λ_a,i,j_* represents *λ* projected after release of biocontrol agent (or combination of biocontrol agents) *a*, by model *i*, under effectiveness combination *j*. This EVPI calculation requires data on all model and effectiveness combinations. However, such a full dataset is not available. Effectiveness levels are not identical for all biocontrol agents (or combination of biocontrol agents) in practice because of real constraints or partial observability. Also, effectiveness data for some biocontrol combinations are not available from field studies. For example, we did not find studies that examined the combination of *T. horridus* and *U. solstitialis*, which makes it impossible to conduct an EVPI analysis including all biocontrol agent combinations. Here, for demonstration purposes, we estimate *λ* under the 25th, 50th and 75th quantiles of the effectiveness levels obtained from the literature survey for each of the three biocontrol agents alone. *Rhinocyllus conicus* and *U. solstitialis* both feed in *C. nutans*' capitula and therefore only affect fecundity. *Trichosirocalus horridus*, on the other hand, feeds on rosettes and can thus also reduce survival and affect growth. However, as there were only two studies that explicitly examined the effect of *T. horridus* on growth and survival, we did not analyse these effects. Therefore, in the above EVPI equation, *a* = 1, 2, …, *A* (*A* = 3), *i* = 1, 2, …, *q* (*q* = 3) and *j* = 1, 2, …, *r* (*r* = 27). The parameter *p_j_* is the weight associated with effectiveness combination *j* (i.e. the belief weight that effectiveness combination *j* is the true effectiveness combination, subject to the constraint that the *p_j_* sum to 1). We assigned equal weight to all models, but these weights could be updated should evidence support a reassessment of model credibility.

We subsequently conducted Expected Value of Partial Information (EVXI; Brand & Small, 1995) analyses to quantify how much the management outcome could be improved by resolving only biological uncertainty or only operational uncertainty. The EVXI analysis considering biological uncertainty as represented by the three demographic models can be quantified as:

(3) $$\text{EVXI}\left(\text{biological}\right)=\left[\sum_{i=1}^{q}p_{i}\min_{a}\sum_{j=1}^{r}p_{j}\lambda_{a,i,j}\right]‐\min_{a}\sum_{i=1}^{q}\sum_{j=1}^{r}p_{i}p_{j}\lambda_{a,i,j},$$

where *n* (*n* = *q* × *r* = 81) model–biocontrol effectiveness combinations are grouped into *i* = 1, …, *q* model sets (*q* = 3). A similar EVXI analysis can be performed for operational uncertainty as represented by the 27 biocontrol effectiveness combinations:

(4) $$\text{EVXI}\left(\text{operational}\right)=\left[\sum_{j=1}^{r}p_{j}\min_{a}\sum_{i=1}^{q}p_{i}\lambda_{a,i,j}\right]‐\min_{a}\sum_{i=1}^{q}\sum_{j=1}^{r}p_{i}p_{j}\lambda_{a,i,j},$$

where *n* model–biocontrol effectiveness combinations are grouped into *j* = 1, …, *r* biocontrol effectiveness combination sets (*r* = 27).

## 3 RESULTS

### 3.1 Population growth rates under a full range of simulated intervention effectiveness

**FIGURE 1:**
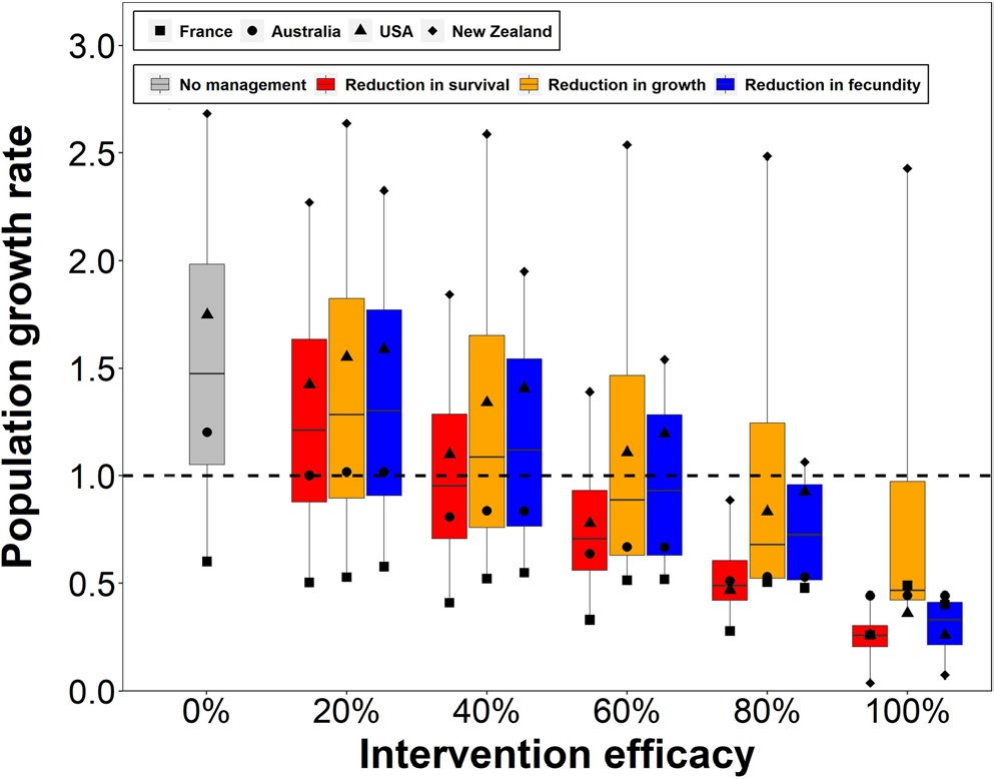
Population growth rate of populations in France, Australia, USA and New Zealand under biocontrol targeting reduction in survival, growth and fecundity. The simulated biocontrol effectiveness ranges from 0% (i.e. without management) to 100%. The interventions were simulated by reducing all relevant demographic rates by the efficacy rate; for example, reducing all survival rates in the matrix model by 20% from their baseline values. The box plots capture the biological uncertainty in the baseline demographic rates as represented by the four populations; the four different types of small dots are the values for the specific populations. The horizontal line towards the middle of the box represents the 50th percentile, while the bottom and top of the box represent the 25th percentile and 75th percentile respectively. See Figure S2 for an alternative visualization that highlights how effectiveness of biocontrol affects different vital rates

In the four populations, the growth rate (*λ*) of *C. nutans* in the absence of management ranges from 0.60 (France) to 2.68 (New Zealand) (Figure 1; Figure S2). Elasticity analysis suggests that thistle growth rate is most sensitive to different parameters in the different populations (Figure S1). Population growth is driven by different demographic processes in different locations, and interventions should therefore target different life‐history vital rates in different contexts (Shea et al., 2005).

Grouping vital rates into three groups (survival, growth and fecundity) and accounting for the effectiveness of intervention, we find that reducing survival results in the largest reduction in *λ* on average, while reducing growth generally ranks second, and reducing fecundity ranks third (Figure 1). However, this ranking is sensitive to the particular model. For example, reducing fecundity performs better than reducing growth for the population in New Zealand, but the opposite is true for the population in USA (Figure S2). Actions' rank may also change when considering the fact that different actions may achieve different effectiveness levels. For example, when both have 20% effectiveness, reducing survival is more effective than reducing growth. However, reducing growth can perform better than reducing survival when it is 40% effective or higher (Figure 1).

### 3.2 Population growth rates under effectiveness levels of biocontrol agents based on empirical studies

**FIGURE 2:**
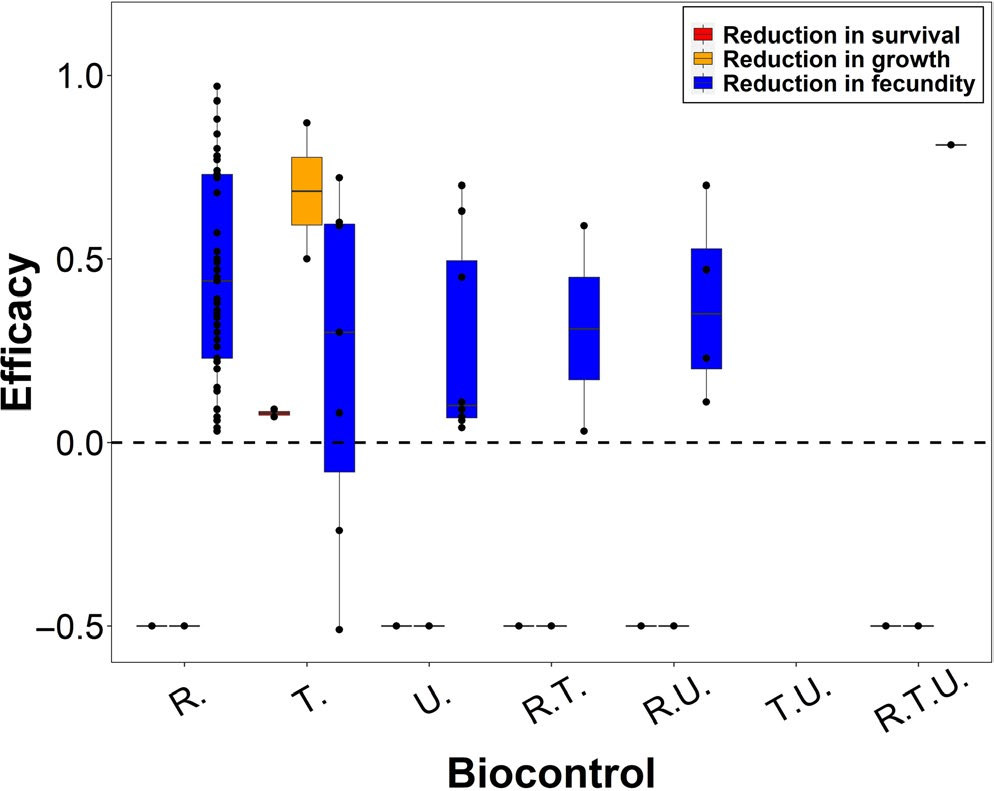
The effectiveness levels of biocontrol in empirical studies based on a literature survey. The biocontrol agents are: *Trichosirocalus horridus* (*T*.), *Rhinocyllus conicus* (*R*.) and *Urophora solstitialis* (*U*.). Combination of agents is shown by combination of the first letters of the agent's genus name, for example, R.T. means the combination of *R. conicus* and *T. horridus*. *Note*: Trichosirocalus horridus may have negative effectiveness, because some results found that Carduus nutans produced more branches (thus potentially more flowers) due to the loss of apical dominance after being attacked by T. horridus. The horizontal dashed line at 0 represents the baseline effectiveness level 0. Black points in each bar represent the effectiveness levels of the corresponding biocontrol agent from empirical studies. The horizontal line towards the middle of the box represents the 50th percentile, while the bottom and top of the box represent the 25th percentile and 75th percentile respectively

Our review of biological control efforts for *C. nutans* identified 17 studies (most with multiple study sites and years) that documented biocontrol effectiveness. Effectiveness varied widely across biocontrol agents (Figure 2), representing a high level of operational uncertainty. *Trichosirocalus* *horridus* is the only biocontrol agent that reduces growth and survival. *Rhinocyllus conicus* achieves the highest effectiveness in reducing fecundity. The combination of *R. conicus* & *U*. *solstitialis* and the combination of *R. conicus* & *T*. *horridus* rank as the second and third most effective in reducing fecundity, respectively (Figure 2). However, the number of records varies greatly across biocontrol agents. *Rhinocyllus* *conicus* is more extensively studied (11 studies) than *T. horridus* or *U. solstitialis* (both with five studies; Figure 2). The effectiveness of biocontrol agents used in combination is far less studied, with only one study on the combination of *R. conicus*, *T. horridus* and *U*. *solstitialis*. The combination of *T. horridus* & *U. solstitialis* has never been assessed in the field (Figure 2). Furthermore, biocontrol agents' effectiveness was only evaluated in a subset of the four countries, except for *R*. *conicus*, which was studied in all four.

**FIGURE 3:**
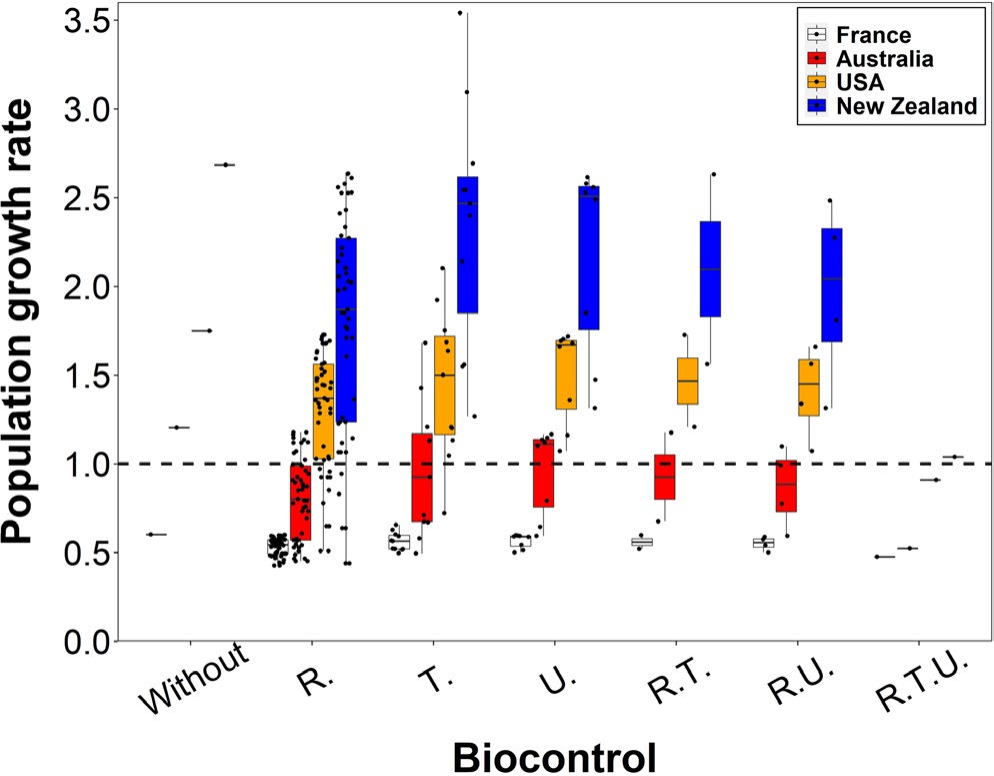
Population growth rate under different effectiveness levels of biocontrol agents in empirical studies. The biocontrol agents are *Trichosirocalus horridus* (*T*.), *Rhinocyllus conicus* (*R*.) and *Urophora solstitialis* (*U*.). Combinations of agents are shown by combination of the first letters of the agent's genus name, for example, R.T. means the combination of *R. conicus* and *T. horridus*

The release of biological control agents is projected to result in different *λ* values in different countries (Figure 3). In the event of a new invasion, where there is uncertainty about which existing invasion is the closest analogue, the models would represent our biological uncertainty. The large variation in *λ* across models represents high biological uncertainty, while the different *λ* values within the same model suggests high uncertainty about control effectiveness. The *λ* values are generally lowest under the full biocontrol agent combination of *R. conicus*, *T. horridus* and *U*. *solstitialis*, and second lowest under the biocontrol agent *R. conicus* (Figure 3).

### 3.3 Value of resolving biological uncertainty in a case study

Value of information analysis focusing on biological uncertainty alone showed that on average in the face of uncertainty, *U. solstitialis* is the optimal single agent with the lowest mean *λ* across countries. This analysis identified *T. horridus* as the optimal single biocontrol agent in Australia and the USA and *U*. *solstitialis* as the optimal in New Zealand. *Rhinocyllus* *conicus* was not optimal in any country (Table 1). The EVPI value was 0.15, which means that when only biological uncertainty is involved, resolving uncertainty could result in a 0.15 reduction in the population growth rate (Table 1).

### 3.4 Value of integrating and resolving both biological and operational uncertainty within the same framework

The full EVPI analysis shows that simultaneous reduction in biological and operational uncertainty would lead to a 15.6% improvement in management outcomes. Additionally, the EVXI analyses conducted within the same framework show that resolving biological uncertainty alone (as represented by the three models in invaded ranges) can improve the management by 8.5%, and resolving operational uncertainty alone (as represented by the 27 biocontrol‐effectiveness‐combinations) can improve management by 10.5%.

In the face of both biological and operational uncertainty, the optimal intervention is to release *T. horridus* (Table 2, last row, last column). In the face of operational uncertainty but with full resolution of biological uncertainty (Table 2, last row), the optimal intervention is either to release *R. conicus* (Australia, USA) or *T. horridus* (New Zealand). In the face of biological uncertainty but with full resolution of operational uncertainty (Table 2, last column), the optimal intervention can be either to release *T. horridus* (17 of 27 effectiveness combinations), *R. conicus* (8 effectiveness combinations) or *U. solstitialis* (2 effectiveness combinations). If all uncertainty can be resolved (Table 2, 27 × 3 cells), any of the three agents might be optimal.

**TABLE 2:**
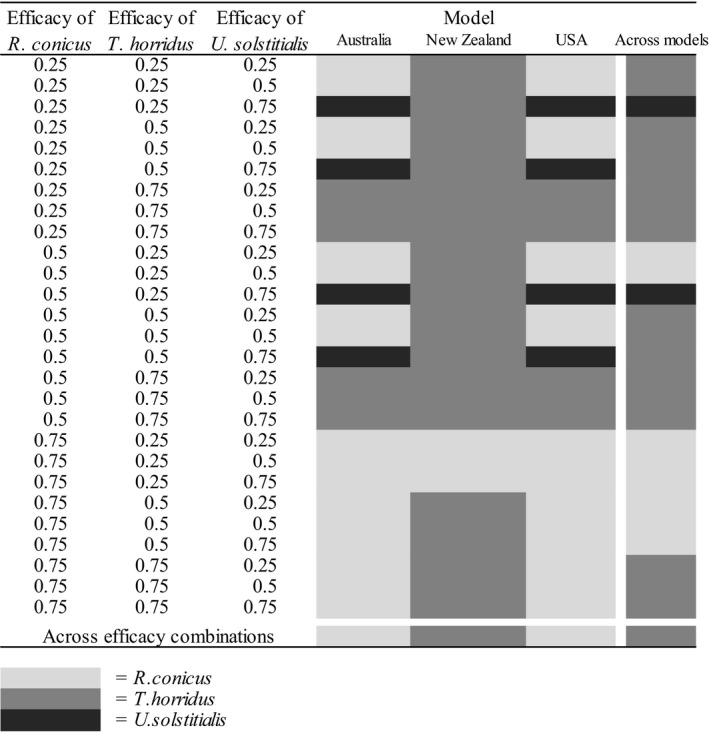
Optimal biocontrol with lowest population growth rate under 27 different biocontrol effectiveness combinations for matrix models of populations in three countries where *C. nutans* is invasive (Australia, New Zealand and USA). The three candidate biocontrol agents are *Rhinocyllus conicus*, *Trichosirocalus horridus* and *Urophora solstitialis*. The effectiveness levels of 0.25, 0.5 and 0.75 represent the 25% quantile, median and 75% quantile of the effectiveness of each biocontrol agent. Cells filled with light grey, intermediate grey and dark grey colour indicate the lowest growth rate under biocontrol by *R. conicus*, *T. horridus* and *U. solstitialis* respectively. The last column and row show the optimal biocontrol agent with lowest population growth rate across models and effectiveness combinations, respectively, while the right bottom cell shows the overall optimal biocontrol agent across models and effectiveness combinations

## 4 DISCUSSION

We used Value of Information analyses to concurrently examine how biological and operational uncertainty affect selection of the most appropriate biological control agent(s) to control *Carduus nutans* in different parts of its invaded range. Our study demonstrates that optimal biocontrol agent selection is jointly determined by the underlying biological processes of the targeted invasive populations and by the effectiveness of candidate biocontrol agents. Ignoring either biological uncertainty or operational uncertainty may result in a suboptimal recommendation. The approach we demonstrate here serves two goals: (a) to identify the control option that is expected to work best given current uncertainties and (b) to estimate how much potential we have to improve management outcomes by conducting research to resolve either type of uncertainty. Using this approach, we show that *T. horridus* is the optimal biological control agent, given biological and operational uncertainty. We further estimate that the musk thistle growth rate could be reduced by 15.6%, on average, if both sources of uncertainty are resolved. However, when either source of uncertainty is ignored, smaller management improvements (8.5% or 10.5%) are achieved (Li et al., 2019).

These findings make it clear that before any biological control agent is released targeting a new invasive population musk thistle, research should be conducted to describe the demography of musk thistle in the novel environment, and to estimate the relative performance of the potential biocontrol agents under local conditions. Both pieces of information have the potential to substantially improve outcomes by identifying the optimal agent for the targeted environment. This recommendation was not a foregone conclusion. If one agent had clearly outperformed the others regardless of the local demography of musk thistle or the range of possible efficacies of the agents, research to determine localized vital rates and the local impact of possible biocontrol agents would be a wasted effort. As a monocarpic perennial, musk thistle has a highly flexible life history, reproducing as an annual, biennial or longer lived perennial depending on local conditions. This flexibility may underlie the importance of gathering demographic information specific to the targeted region for appropriate biocontrol agent selection. As managers working in other systems confront similar decisions, recommendations about whether to carry out additional studies or to move ahead with management will depend on details of the focal system.

The framework we present here is flexible, and can be adapted to include alternative methods for encapsulating uncertainty. In our study, biological uncertainty is represented by multiple models of populations from different locations across the invaded range. Alternatively, biological uncertainty could be incorporated by including variation around each parameter in the demographic model (Moore & Runge, 2012). Biological uncertainty can also be represented by considering models with different structures (e.g. Li et al., 2017) or models applying different modelling approaches, such as individual‐based models versus population‐based models (Shea et al., 2005; Shea et al., 2006). Uncertainties associated with more nuanced aspects of the dynamics, such as nonlinearities, context‐dependent efficacy and interactions among interventions, can also be evaluated with this framework. To make these types of model syntheses possible, we encourage demographic modellers and researchers reporting management impacts to present their work in a way that can be easily used by researchers with related research interests. For example, for a matrix model, instead of only presenting the overall transition values, it can be helpful to list the vital rates that make up the transition values (Jongejans et al., 2008).

Operational uncertainty is rarely quantified, but our results indicate that it is no less important than biological uncertainty. Our EVPI analysis shows that resolving operational uncertainty can improve the management outcome by up to 10.5% (Table 2). Even in our well‐studied system, there are still some combinations of biological control agents that have not been studied or are poorly addressed. For example, our literature survey did not identify studies that combined *T. horridus* and *U. solstitialis*, even though these are the two most effective biocontrol agents in our simple EVPI example (Table 1; Shea et al., 2005). Furthermore, our models suggest that these agents may be highly effective when released together. It could be instructive to test our prediction with this combination should the data become available. In addition to evaluating the mean effectiveness of management options, decision makers should also consider variation in effectiveness. For example, a control with a better mean effectiveness but also a large variation in effectiveness may not necessarily be preferred over one with a lower mean but also a smaller variation, especially in situations where managers are risk averse.

In order to highlight the importance of both biological and operational uncertainty, we focused on a study system where extensive prior work has provided data that may be used to represent these two types of uncertainty. However, biocontrol practitioners or managers of other invasive species do not need to have a similar quantity of data to employ this decision‐making framework. When confronted by an invasion requiring management, regardless of the amount of information available, the framework illustrated in this study can be applied through the following procedure to assist the decision‐making process: (a) Identify the management objective (our case study objective is the reduction of musk thistle population growth rate, but it could be many other metrics in other settings, e.g. Shea et al., 2005; Probert et al., 2016) and alternative interventions (we examine alternative biocontrol agents, but any sort of intervention, such as mowing, crash grazing or herbicide spraying, is possible) for achieving that objective. (b) Synthesize existing knowledge in a model that is appropriate for the decision setting and the species in question. We used a matrix model based on life‐history states, but other model structures would be valuable in other settings. While models must provide comparable metrics of the objective, they may otherwise take very different forms. Where data are not available, expert judgement processes would be appropriate to estimate parameters for the model (Runge et al., 2011). (c) Consider key sources of uncertainty in the models. Biological uncertainty is represented well by the variety of choices in selecting model structure and in estimating demographic parameters. A variety of estimates for candidate management action impacts can capture operational uncertainty. (d) Use VoI tools to identify which of the sources of uncertainty, if any, are impediments to making a decision. If recommendations are clear, despite uncertainty, management may proceed. If decisions depend on a key uncertainty, the VoI analysis quantifies the benefit of learning before a decision is made, allowing the manager to prioritize research that will help most with management. In the case of biological control release, a species release either occurs or it does not, but in other pest management decision settings, repeated decisions may be made as information is gained. An adaptive management approach, in which management and research are conducted concurrently in a formal framework, may be warranted in such cases (Williams & Johnson, 1995).

Overall, our study demonstrates that optimal invasion control can be jointly affected by biological and operational uncertainty. Here, we illustrate a Value of Information framework to integrate multiple biological models and management effectiveness combinations. In this framework, the effects of biological uncertainty and operational uncertainty on management outcomes can be evaluated. This analysis is especially important when research effort or time is limited, as in disease outbreaks (Li et al., 2017), or in efforts to eradicate invasive species after early detection before they spread in the invaded range (Moore, Runge, Webber, & Wilson, 2011). Additionally, EVXI analysis, which quantifies how much the management outcome can be improved by resolving a certain source of uncertainty, can guide the design of the most appropriate information collection strategy. The framework presented in the current study will be especially valuable in systems with significant uncertainty.

## AUTHORS' CONTRIBUTIONS

S.‐L.L., J.K., M.C.R. and K.S. conceived the ideas and designed the methodology; S.‐L.L. and K.S. collected the data and analysed the data; S.‐L.L., J.K., M.C.R. and K.S. wrote the manuscript. All authors contributed critically to manuscript drafts and gave final approval for publication.

## Supporting information

Fig S1‐S2

Table S1‐S8

## Data Availability

Data available via the Dryad Digital Repository https://doi.org/10.5061/dryad.83bk3j9rf (Li et al., 2021).
