# Supplementary material for: Weighing the unknowns: Value of Information for biological and operational uncertainty in invasion management
Source: J Appl Ecol. 2021 Jun 22;58(8):1621–30. doi: 10.1111/1365-2664.13904 (PMC8453580; doi:10.1111/1365-2664.13904)
Supplement: Supplementary file 1 — Fig S1‐S2 [file JPE-58-1621-s001.docx]

*
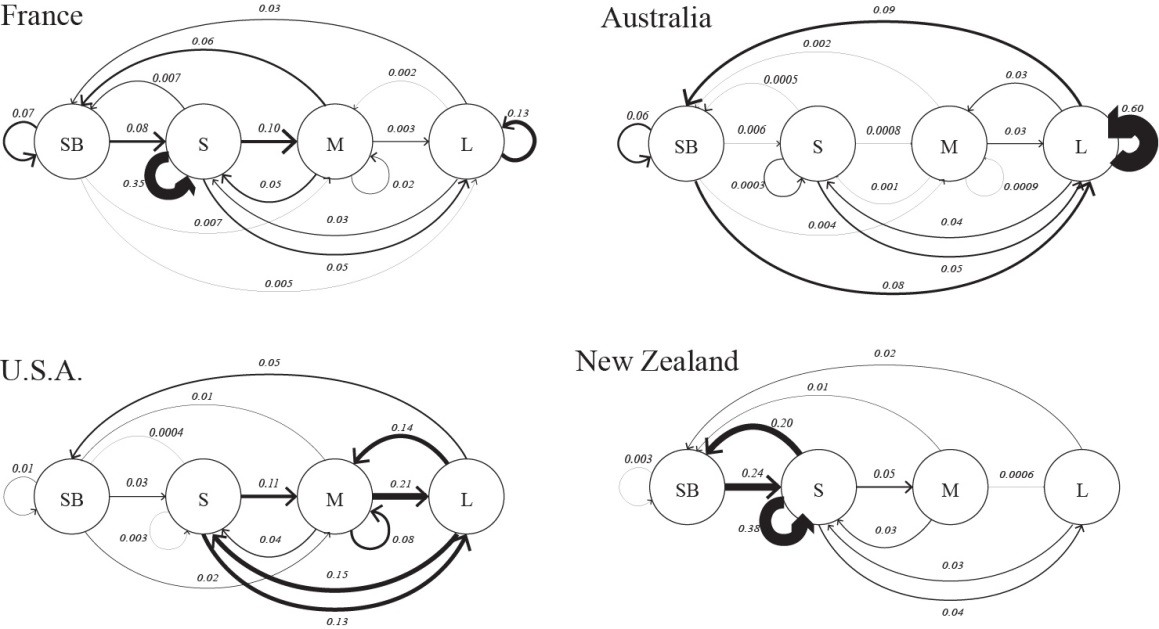
*

Figure S1. Life cycle diagram with elasticity values of transitions between life stages of *Carduus nutans*. The matrices of the four populations include one in the native range, France, and three in the invaded ranges, which are Australia, USA, and New Zealand. Biocontrol agents were present in the native population in France, but not in the invaded range populations in Australia, USA, and New Zealand. The four life stages are seed bank (SB), small rosettes (S) with <20% probability of surviving and flowering in the next year, medium rosettes (M), and large rosettes (L) with >80% chance of surviving and flowering next year. The thickness of the transition arrows from thin to thick represents the elasticity values of life history transitions from low to high.


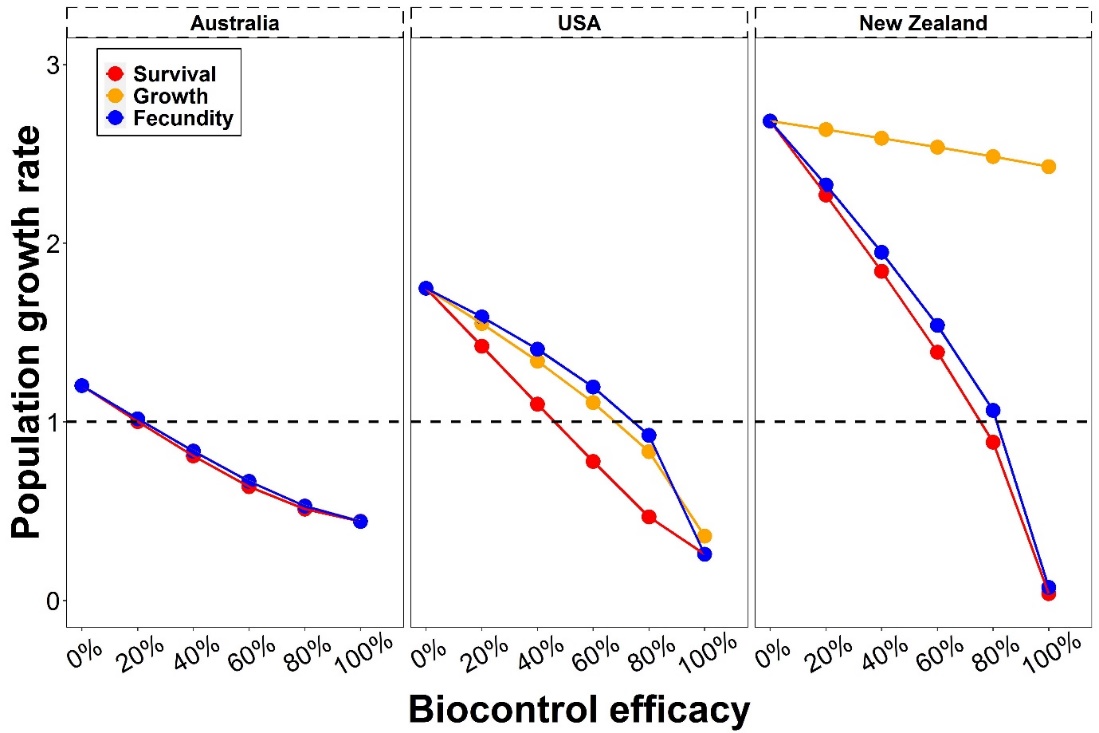


Figure S2. Projected population growth rates of populations in Australia, the USA, and New Zealand under biocontrol targeting reduction in survival, growth and fecundity. The simulated biocontrol effectiveness ranges from 0% (i.e., without management) to 100% with an interval of 20%.
